# Promoting Effect of Palladium on ZnAl_2_O_4_-Supported Catalysts Based on Cobalt or Copper Oxide on the Activity for the Total Propene Oxidation

**DOI:** 10.3390/ma14174814

**Published:** 2021-08-25

**Authors:** Marco Antonio Ocsachoque, María Silvia Leguizamón-Aparicio, Mónica Laura Casella, Ileana Daniela Lick

**Affiliations:** CINDECA (CCT La Plata-CONICET-UNLP), Departamento de Química, Facultad de Ciencias Exactas, Universidad Nacional de La Plata, Calle 47 N° 257, La Plata, Buenos Aires 1900, Argentina; ocmarco@quimica.unlp.edu.ar (M.A.O.); mariasilvialap@quimica.unlp.edu.ar (M.S.L.-A.); casella@quimica.unlp.edu.ar (M.L.C.)

**Keywords:** zinc aluminate, palladium, cobalt oxide, copper oxide, propene catalytic oxidation

## Abstract

Palladium-modified Co-ZnAland Cu-ZnAl materials were used and found active for the catalytic oxidation of propene and propane. According to the results obtained by XRD, TPR and XPS, the zinc aluminate-supported phases are oxide phases, Co_3_O_4_, CuO and PdO_x_ for Co-ZnAl, Cu-ZnAl and Pd-ZnAl catalysts, respectively. These reducible oxide species present good catalytic activity for the oxidation reactions. The addition of palladium to Co-ZnAl or Cu-ZnAl samples promoted the reducibility of the system and, consequently, produced a synergic effect which enhanced the activity for the propene oxidation. The PdCo-ZnAl sample was the most active and exhibited highly dispersed PdO_x_ particles and surface structural defects. In addition, it exhibited good catalytic stability. The H_2_ pre-treated PdCu-ZnAl, PdCo-ZnAl and Pd-ZnAl samples showed higher activity than the original oxide catalysts, evidencing the important role of the oxidation state of the species, mainly of the palladium species, on the catalytic activity for the propene combustion. The synergic effect between metal transition oxides and PdO_x_ could not be observed for the propane oxidation.

## 1. Introduction

Air pollution represents a significant risk to human health. This risk is due to human exposure to toxic pollutants present in the atmosphere, which can cause diseases such as pneumonia, chronic bronchitis and lung cancer. In 2019, the World Health Organization reported that air pollution causes millions of deaths each year [1,2]. Volatile organic compounds (VOCs) are formed of a complex mixture of hundreds of gases that contain carbon, excluding carbon monoxide, carbon dioxide, carbonic acid, metallic carbides or carbonates and ammonium carbonate [3], and can be mentioned among the main atmospheric pollutants. VOCs are generated from several sources, such as industrial processes (e.g., petroleum refining, textile dyeing and printing, etc) and mobile sources (e.g., vehicle engines). Among the compounds called VOCs are saturated alkanes, unsaturated alkenes and alkines, aromatic hydrocarbons, oxygen-containing VOCs and chlorinated VOCs. These compounds can participate in photochemical reactions, in the atmosphere, producing ozone and ultrafine particles that make up photochemical smog [4].The most reactive molecules in this type of photochemical reactions are those that have a C=C double bond in their structure since they allow radicals to be easily added [5]. Therefore, the VOC emission to the atmosphere is an environmental problem that must be solved. In this sense, various processes have been proposed for the elimination of VOCs, such as adsorption on activated carbon, cryogenic condensation, chemical absorption and total catalytic oxidation. The latter being one of the most promising processes [6]. Among the most difficult pollutants to remove by deep catalytic oxidation are the short-chain saturated and unsaturated hydrocarbons, also called remaining hydrocarbons, which are present in automobile exhaust pipes emissions.

The deep catalytic oxidation, also called combustion, of remaining hydrocarbons has been studied extensively in recent decades. In general, the most used model molecules to study the elimination of short-chain hydrocarbons were those containing low carbon number (<C6) and, particularly, propane and propene (C3). A great variety of catalysts have been reported as active for these oxidations and, to date, numerous works and bibliographic reviews that analyze the state of the art of the subject, have been published [7,8,9,10,11,12,13,14,15,16,17,18,19,20,21,22,23,24,25]. These reports include the well know active systems based on the use of supported noble metals (palladium, platinum, ruthenium, gold and rhodium, among others), and systems based on the use of oxide compounds (transition metal oxides, spinels, derivative oxides hydrotalcites, etc.), which, recently, have been studied more extensively.

The noble metal-containing catalysts, and particularly those that contain Pt and Pd, exhibit high activity but present some disadvantages: mainly high cost and low availability [7,8,9]. Research has focused on minimizing their use or replacing them with oxide catalysts. Although there are numerous bibliographic reports of non-noble metals based catalysts, the bulk transition metal oxides (TMOC) and the supported transition metal oxides catalysts (STMOC) have been found to be very active, mainly those based on the use of Co_3_O_4_, CuO and MnO_x_, among others [11,12,13,14,15,16,17,18,19,20,21,22,26].

The Co_3_O_4_, bulk or supported, is considered one of the more promising active oxides, either for the combustion of saturated, unsaturated or polyaromatic hydrocarbons. Its activity in the deep propane oxidation has been widely reported and, also, to a lesser extent, there are reports that show activity for the propene combustion [11,12,13,14,15,16]. The high activity of this oxide is mainly associated with its redox properties and its lattice oxygen mobility, which can be modified with a suitable choice of the support [17,18]. Another promising active phase is copper oxide, CuO, which has been less studied for this application, but it is known that the addition of CuO to other oxide systems, such as Co_3_O_4_ or MnO_x_ increases the activity [19,20,21,22,27].

On the other hand, more recent studies focused on the study of the synergy between metallic and non-metallic phases. In some catalytic systems, the addition of small amounts of noble metals (Pt, Pd, Au and Rh) produced an increase in the activity of TMOC and STMOC [28,29,30,31,32,33,34,35]. For example, Kamiuchi et al. reported that the addition of palladium to ceria-zirconia oxide produces an increase in propene combustion [28].In addition, a promotional effect of CeO_2_ on palladium supported on alumina pillared clays was found for the deep propene oxidation [36]. Liotta et al. showed that a suitable interaction between supported gold nanoparticles and an oxide support not only improved the catalytic activity of the catalysts for the oxidation of methane, but also improved the catalytic stability [37]. Solsona et al. found that the addition of gold to Co_3_O_4_–containing catalysts produced an increase in the system reducibility and the oxygen mobility and, consequently, the activity for oxidation reactions is promoted [38]. Similar effects were found in previous works of our working group in which it has been shown that the addition of noble metals (Au, Rh) to cobalt oxide (Co_3_O_4_) containing catalysts produced an activity increase. For example, the addition of gold to a Co_3_O_4_/tetragonal zirconia catalyst generated an increase in the activity for the combustion of propane and naphthalene and the addition of rhodium to the Co_3_O_4_/zinc aluminate system promoted the activity for the deep propane oxidation [29,31].

In addition to the active phase, the nature of the support is another aspect to consider. Generally, the support has an influence on the catalytic activity due to its textural, acid base and redox properties. Although among the most reported supports for these reactions are CeO_2_, ZrO_2_ and Al_2_O_3_, however, in recent years has appeared great interest in the use of zinc aluminate, ZnAl_2_O_4_. This oxide has the particularity of presenting great thermal and chemical stability, certain hardness and high specific surface [39,40]. On the other hand, it has been reported that zinc aluminate presents a strong support metal interaction with noble metals, which can favor the stability of the metallic particles [41]. This low-cost support presents the complete spinel structure, which prevents the diffusion of the supported species in the support network. In a previous work, it has been shown that rhodium-Co_3_O_4_active phases supported on zinc aluminate exhibited higher activity than a similar alumina-supported catalyst. Zinc aluminate as support leads to a great availability and activity of surface phases [29]. On this basis, this work shows results of cobalt oxide catalysts and copper oxide catalysts supported on non-commercial ZnAl_2_O_4_ for the catalytic combustion of propene, as a model molecule for short-chain unsaturated hydrocarbons. The promoting effect of a small amount of palladium (0.5 wt.%) added to these catalysts is particularly studied. Additionally, the effect of the oxidation state of palladium on activity and some aspects of the reaction mechanism were analyzed. Finally, the prepared catalysts were also tested in the oxidation reaction of propane, a saturated hydrocarbon.

## 2. Materials and Methods

### 2.1. Preparation of Support and Catalysts

Zinc aluminate (ZnAl_2_O_4_) was prepared with the coprecipitacion method, from Zn(NO_3_)_2_·6H_2_O (Sigma-Aldrich, Argentina) and Al(NO_3_)_3_·9H_2_O (Sigma-Aldrich, Argentina) solutions, in ammoniacal medium (pH = 10), following the procedure described elsewhere [29]. This support, labeled ZnAl, was obtained by calcinating at 600 °C. Portions of ZnAl were impregnated with an aqueous solution of Cu or Co nitrates in an ammoniacal medium to obtain materials with 5 wt.% metal concentrations [42]. After being continuously stirred for 6 h, the mixtures were filtered, dried and calcined at 600 °C for 1 h. The monometallic palladium catalyst was prepared by impregnating the zinc aluminate with an aqueous solution of PdCl_2_ (Sigma-Aldrich, Argentina) to obtain catalysts with a nominal palladium content of 0.5 wt.%. After drying, the sample was calcined under an O_2_ flow rate of 60 mL/min at 500 °C for 1 h. Finally, the bimetallic catalysts, containing 0.5 wt.% Pd, were prepared by impregnation of CoO_x_ and/or CuO_x_ supported on ZnAl_2_O_4_. Then, the samples obtained were dried and subsequently calcined in O_2_ at 500 °C for 1h. The catalysts obtained were labeled as Cu-ZnAl, Co-ZnAl, Pd-ZnAl, PdCu-ZnAl and PdCo-ZnAl.

To study the effect of the palladium oxidation state on the catalytic activity, palladium containing catalysts were thermally treated at 160 °C for 1h in a reducing atmosphere (H_2_ 10 vol.%/N_2_). These samples were labeled as Pd-ZnAl_red_, PdCu-ZnAl_red_ and PdCo-ZnAl_red_.

### 2.2. Catalyst Characterization

The catalysts were characterized by several physicochemical techniques. The N_2_adsorption–desorption isotherms were obtained using a Micromeritics ASAP 2020 instrument (Micromeritics, Norcross, GA, USA) at −196 °C. Before each measurement, the samples were degassed at 100 °C for 12 h. The specific surface areas were determined by the Brunauer–Emmett-Teller (BET) method.

The crystalline phases were identified by X-ray powder diffraction (XRD) on an X-ray diffractometer (Philips PW 1740, Philips, Eindhoven, The Netherlands) with Cu Kα (Ni-filtered) radiation operated at 20 mA and 40 kV.

In a typical temperature-programmed reduction (H_2_-TPR) experiment, the sample (0.1 mg) was placed in an electrically heated fixed-bed quartz micro-reactor, and heated from 25 to 900 °C at a heating rate of 10 °C/min, employing a 10% (*v*/*v*) H_2_/N_2_ (flow rate 20 mL/min) gas mixture.

Scanning electron microscopy with energy-dispersive X-ray spectroscopy (SEM-EDS) analysis was performed in order to obtain elemental mapping and semi-quantitative analyses by using a FEI ESEM Quanta 200 equipment (Thermo Fisher Scientific, formerly FEI Company, Hillsboro, OR, USA).

(1) $$d_{AV}=\frac{\sum n_{i}d_{i}^{3}}{\sum n_{i}d_{i}^{2}}$$

where *n_i_* is the number of particles of *d_i_* size.

Chemical states of cobalt, copper and palladium supported species were investigated by X-ray photoelectron spectroscopy (XPS) on a Physical Electronics PHI-5700 spectrometer, using Al Kα radiation (1486.6 eV). XPS data were calibrated using the binding energy of C*1s* (284.8 eV) as the standard. The Casa XPS program was used for the analysis of the data. Cobalt, copper and palladium spectra were deconvoluted using the least squares fitting routine incorporated in this software with a Gaussian/Lorentzian (30/70) product function, after subtraction of a non-linear baseline. The peak areas obtained were used to determine the surface composition and the sensitivity factors method was applied [43].

### 2.3. Catalytic Activity

Propene combustion was carried out using an electrically heated fixed-bed quartz reactor containing 0.100 g of catalyst. The catalytic activity was studied with a gaseous mixture feed containing 1000 ppm of C_3_H_6_, 6 vol.% O_2_ and He to close the balance (total flow rate = 50 mL/min).

The catalytic activity for propane oxidation was carried out in a fixed bed quartz reactor containing 0.100 g of catalyst with a feed mixture consisting of C_3_H_8_/He, O_2_/He and He to close the balance. The reaction flow contained 1000 ppm C_3_H_8_ and 8% *v*/*v* O_2_. The total gas flow rate was 50 mL/min.

In both processes, the reactant and reaction products streams were analyzed online using a Shimadzu CG 2014 (Shimadzu Corporation, Kyoto, Japan) provided with a TCD. Conversions of C_3_H_6_ and C_3_H_8_ to CO_2_ were determined from the area of the CO_2_ peaks obtained chromatographically. The separation of products was performed with Porapack Q and 5 A molecular sieve columns. The temperature of the catalytic bed was monitored by a k-type thermocouple and the temperature was varied from 150 °C to 600 °C at 1.6 °C/min. A schematic diagram of the reaction systems has been reported in a previous work [44].

The experiments of propene activation were performed in the absence of oxygen, under the same condition adopted for the experiments of propene oxidation by using an on-line mass spectrometer (Dycor, Dymaxion Analyzer, Ametek Process Instruments, Newark, DE, USA) following the most intense molecular ion peaks of C_3_H_6_ (*m*/*z* = 41), CO_2_ (*m*/*z* = 44) and H_2_ (*m*/*z* = 2).

## 3. Results and Discussion

### 3.1. Characterization of Catalytic Materials

#### 3.1.1. Morphological Characteristics of the Catalysts

Both the support and the catalysts were characterized by N_2_ adsorption/desorption, and the experimental data were adjusted by the BET method. All the samples, monometallic and bimetallic catalysts, exhibited type IV isotherms with H1 hysteresis cycle, according to IUPAC. These results indicate that the samples are mesoporous [45]. Figure S1 (Supplementary Material) shows, as an example, the adsorption and desorption isotherms of the support and the PdCo-ZnAl catalyst.

The zinc aluminate support presents a BET area of 50 m^2^/g and a pore volume of 0.280 cm^3^/g. Cu-ZnAl, Co-ZnAl, Pd-ZnAl and CuPd-ZnAl catalysts show specific surface area and pore volume values such as those observed for the support (Table 1). Instead, the PdCo-ZnAl sample shows a lower specific surface. This fact could be associated with a covering of the pores of the support by new species of cobalt and/or palladium formed during the calcination treatment.

From the desorption isotherm of each sample, the pore diameter (Table 1) was calculated using the BJH method. The results obtained indicate that the addition of the active phases to the support does not lead to a significant modification in the pore diameter of the support.

#### 3.1.2. X-ray Diffraction (XRD)

**Figure 1:**
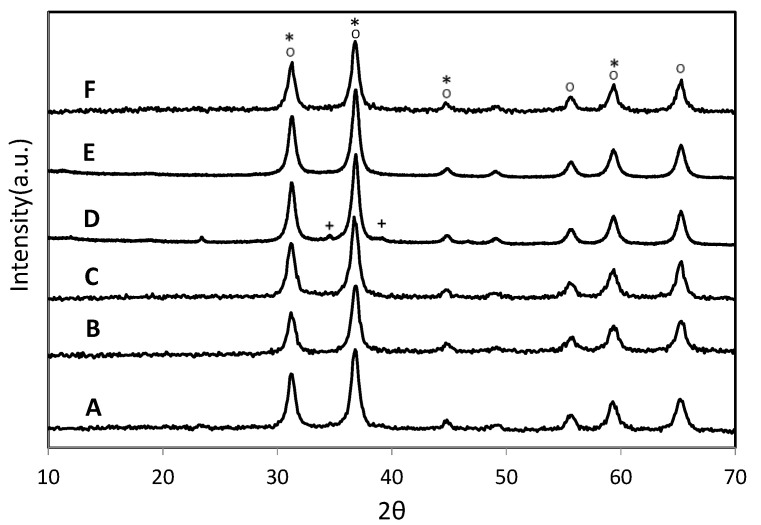
XRD diffractograms of catalysts. (A) ZnAl; (B) Co-ZnAl; (C) Pd-ZnAl; (D) Cu-ZnAl; (E) PdCu-ZnAl and (F) PdCo-ZnAl. ° ZnAl_2_O_4_, * Co_3_O_4_ and ^+^ CuO.

Powder X-ray diffraction studies were performed to investigate the crystalline phases of both the support and the active species. XRD profiles are presented in Figure 1. All the samples exhibit diffraction lines located at 2θ: 31.29, 36.86, 44.83, 59.39 and 65.27° (PDF 03-065-3104) typical of ZnAl_2_O_4_ [39].

Neither signals due to ZnO (2θ = 32.01, 34.65, 36.49, 47.77, 56.79, 63.02, 66.59, 68.10 and 69.23°, JCPDS N° 75–1526) were detected, nor signals associated with palladium species, PdO (2θ = 33.60, 33.90, 41.96, 54.84, 60.25 and 60.86°, JCPDS N° 06–0515) nor Pd(0) (2θ = 39.7, 46.5 and 67.9°, JCPDS N° 05–0681). These last diffraction lines cannot be detected because the palladium was added in a very low concentration and the crystallite size of its species is under the resolution of the equipment used.

The diffraction lines of the Co_3_O_4_ spinel (2θ = 31.2, 36.8, 59.3 and 65.1°, PDF N° 01-080–1533) are difficult to distinguish from those exhibited by the support since both compounds exhibit the same crystalline structure.

The presence of CuO is poorly evidenced in the diffraction profile obtainedfor the Cu-ZnAlsample, withsmall peaks located at 2θ = 35.5 and 38.7° (PDF N° 01-080-1917). However, these signals cannot be evidenced in the XRD pattern of CuPd-ZnAl sample.

#### 3.1.3. Temperature Programmed Reduction (TPR)

**Figure 2:**
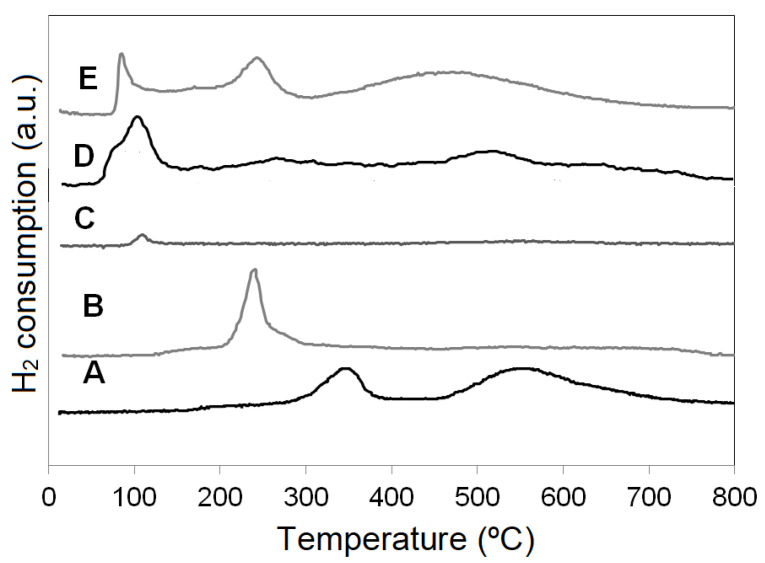
TPR profiles of catalysts. (A) Co-ZnAl; (B) Cu-ZnAl; (C) Pd-ZnAl; (D) PdCu-ZnAl; (E) PdCo-ZnAl.

To analyze the presence of oxide phases, including those not revealed by XRD, and the palladium addition effect on reducibility, temperature programmed reduction (TPR) analysis wasperformed. Figure 2 shows the H_2_ consumption as a function of temperature for the studied catalysts. In the TPR profile of Pd-ZnAl catalyst (Figure 2, curve C), a low intensity signal at approximately 100 °C can be observed. This signal is assigned to the reduction ofPdO_x_ [28,46].

As it has been reported in a previous work, in the TPR profile of Co-ZnAl catalyst (Figure 2, curve A) two reduction zones can be observed. The first zone, located at temperatures below 400 °C, presents a reduction signal centered around 350 °C and associated with the reduction of Co_3_O_4_ supported species, weakly interacting with the support. The reduction signal located above 400 °C, centered around 550 °C, is associated with the reduction of cobalt species with a higher interaction with the support [29,47].

The PdCo-ZnAl bimetallic catalyst (Figure 2, curve E) shows H_2_ consumption signals starting from 70 °C. The first signal (at ~84 °C) can be associated with the reduction of the oxidized palladium species (PdO_x_) and/or the reduction of cobalt oxide species that are in the vicinity and/or in a very close interaction with the palladium oxide. The presence of Pd(0), generated during the reduction, favors the H_2_ spillover and, consequently, generates an increase in the reducibility of the rest of the cobalt oxide species and of those cobalt ionic species with greater interaction with the support, which show reduction signals centered at 242 °C and 458 °C, respectively. It is important to note that the reduction signal assigned to the reduction of palladium also shifts towards a lower temperature than that observed for the Pd-ZnAl catalyst, indicating that in this sample the PdO_x_ species are more dispersed or more available in the catalytic surface.

The Cu-ZnAl catalyst shows (Figure 2, curve B) a reduction signal centered around 240 °C, associated with the reduction of CuO [48], while the PdCu-ZnAl catalyst (Figure 2, curve D) shows a similar behavior to that of the PdCo-ZnAl catalyst. The addition of palladium to Cu-ZnAl catalysts also increases the reducibility of the system. The TPR diagram is dominated by a signal centered at 130 °C, associated with the reduction of dispersed palladium oxide species and CuO. For this system, the reducibility is also favored by the H_2_ spillover and, furthermore, it cannot be ruled out that the addition of palladium modified the dispersion or nature of the copper oxide species. Generally, the smaller copper oxide species reduce at a lower temperature. In this context, it should be clarified that, according to the results obtained by XRD, the oxide species present in the PdCu-ZnAl catalyst have smaller crystalline size than those observed in the Cu-ZnAl catalysts. In addition, at higher temperatures, low intensity signals appear, indicating the presence of some copper species with greater interaction with the support [49,50].

The TPR profiles of palladium containing samples do not present a negative peak around 70 °C, which is associated with the decomposition of the β-PdH phase. This signal is usually observed in TPR profiles of samples that contain palladium metallic species [28]. This fact reinforces the hypothesis of the presence of palladium oxide species.

#### 3.1.4. Scanning Electron Microscopy (SEM) and Energy Dispersive Spectroscopy (EDS)

In order to analyze the morphology and the distribution of the active phases on the catalytic surface, SEM micrographs and EDS elemental mapping were performed. Figure S2Aa–e (Supplementary Material) show SEM images, while Figure S2Ba–j (Supplementary Material) show the Co or Cu, Pd, Al and Zn elemental mapping obtained for each catalyst.

The morphology of catalysts depends on the supported phases, cobalt containing samples (Co-ZnAl or PdCoZnAl) presented larger particles than those found in the Pd-ZnAl sample or copper containing samples (Cu-ZnAl or PdCu-ZnAl).

Elemental mapping images, obtained using magnifications of 300× and 5000×, showed homogeneously dispersed shining points for each element for all the catalysts analyzed. These results suggest that the supported active phases are well distributed and dispersed on the catalytic surface.

SEM-EDS tests were also performed with the purpose of obtaining a semi-quantitative analysis of palladium, copper and cobalt content. The calculated Co/Al, Cu/Al or Pd/Al atomic ratios are listed in Table 2. These atomic ratios obtained are quite higher than the nominal ones, suggesting that the supported oxides are mostly exposed on the catalytic surface.

#### 3.1.5. X-ray Photoelectron Spectroscopy (XPS)

XPS analysis of catalysts was performed to provide information both on the chemical state of the elements and on the surface chemical composition of the studied catalysts.

For the support (Figure S3, Supplementary Material) and all catalysts, the Al 2*p* core level spectrum showed a peak located at 74.4 eV (B.E.) while the Zn 2*p* core level spectrum exhibited a doublet with binding energies at 1022.7 eV and at 1045.8 eV, attributed to the Zn 2*p*_3/2_ and Zn 2*p*_1/2_ components, respectively. The O 1*s* core level spectrum is composed of two components at about 531.6 and at 533.2 eV, attributed to lattice oxygen species, mostly from the support, and adsorbed molecular water, respectively [29]. In agreement with previously reported results for rhodium promoted Co_3_O_4_/ZnAl_2_O_4_ catalysts, the Al 2*p* Zn 2*p* and O 1*s* core levels spectra are typical of ZnAl_2_O_4_ [29,51].

Palladium-containing catalysts displayed the Pd 3*d* core level spectra with doublets corresponding to Pd 3*d*_5/2_ and Pd 3*d*_3/2_, at 337 and 342 eV, respectively, attributed to Pd(II) species [52,53,54]. Figure S4 (Supplementary material) shows the Pd 3*d* spectra of catalysts and Table 3 shows the binding energy (B.E.) obtained.

Both cobalt-containing catalysts, Co-ZnAl and PdCo-ZnAl, displayed the Co 2*p* core level spectra with doublets corresponding to Co 2*p*_3/2_ and Co 2*p*_1/2_ at ~781 and ~796 eV (Table 3), respectively, separated by spin orbit splitting energy of ~15 eV. Both signals exhibited their respective shake-up satellites. The presence of the satellite corresponding to the Co 2*p*_3/2_ core level is associated with the presence of Co^2+^ paramagnetic species. Moreover, the Co 2*p*_3/2_ spectrum is composed of two bands centered at ~780.3 and ~782 eV attributed to Co^3+^ and Co^2+^, respectively. Figure S5 (Supplementary Material) shows the Co2*p* XPS spectra and the deconvolutions, corresponding to Co-ZnAl and PdCo-ZnAl catalysts. The results obtained suggest the presence of the Co_3_O_4_ spinel [55,56,57,58].

**Figure 3:**
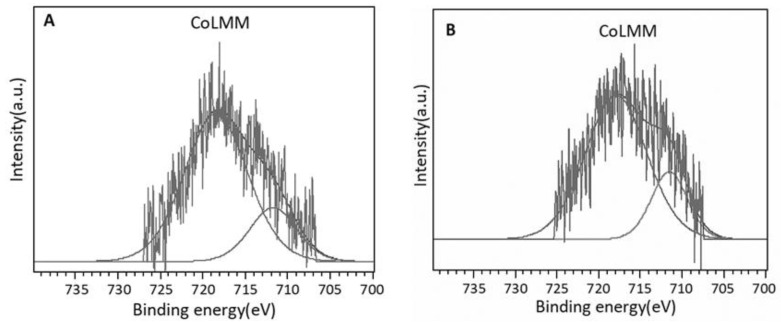
CoLMM Auger region spectra and deconvolutions: (**A**) Co-ZnAl; (**B**) PdCo-ZnAl.

To obtain more precise information about the oxidation states of the cobalt supported species, the CoLMM Auger signals were also analyzed. The chemical shifts of the Auger peaks are generally greater than the photoelectronic ones. Figure 3 shows the spectra in the CoLMM Auger region and Table 3 presents the Co2*p*_3/2_ peak binding energies (B.E.) and the kinetic energy (KE) of the CoLMM Auger transition. In both samples, modified Auger parameter (AP *) values of ~1549 and 1555 eV were obtained, which are associated with the presence of Co(II) and Co(III) species, respectively [59].

Furthermore, the addition of palladium modifies the chemical environment of cobalt, since the B.E. of these species shifts towards higher values, probably due to the formation of Co-Pd interaction compounds. Likewise, a slight modification of the cobalt oxidation states is also observed, the PdCo-ZnAl catalyst has a higher Co(II)/Co(III) atomic ratio. In this context it is important to note that the values of the Co(II)/Co(III) atomic ratios are higher than the one expected for Co_3_O_4_-like species for which the atomic ratio is equal to 0.5. These results suggest that the Co^2+^ content originates both from Co_3_O_4_ spinel structures where the Co(II) ions are tetrahedrally coordinated and from Co(II) ionic species spread on the surface, thereby increasing the Co^2+^/Co^3+^ ratio. Similar results have been found with rhodium promoted cobalt supported catalysts [29].

The XPS spectra of the Cu2*p* region collected on unpromoted and Pd-promoted catalyst are illustrated in Figure S6 (Supplementary Material). The Cu2*p* core level spectra of Cu-ZnAl and PdCu-ZnAl samples exhibited signals associated with the presence of Cu(II) species on the catalytic surface. In this sense, both catalysts displayed spectra with doublets corresponding to Cu2*p*_3/2_ andCu 2*p*_1/2_, at 933.5-933.7 eV and 953.3-953.4 eV [60,61]. These signals exhibit their respective shake-up satellites (Figure S6) that are also associated with the Cu(II) presence [61]. However, the presence of Cu(I) species cannot be completely ruled out, pure CuO exhibits a I_sat_ (intensity of satellite) to a I_mp_ (intensity of the main peak) ratio of 0.55 while the I_sat_/I_mp_ ratios found for the Cu-ZnAl and PdCu-ZnAl catalysts were below than the theoretical one. On the other hand, the addition of palladium to the Cu-ZnAl catalyst generates a slight shift of the B.E. of copper towards higher values, evidencing the formation of interaction species [62].

The surface atomic composition was analyzed by integrating the XPS peak areas of the Cu, Co, Pd and Al elements, using the sensitivity factors method [43]. Table 2 summarizes the Co/Al, Cu/Al and Pd/Al surface atomic ratios. The addition of palladium to the Co-ZnAl catalyst causes a decrease in the surface cobalt content. A similar behavior is observed with the addition of palladium to the Cu-ZnAl catalyst. On the other hand, the PdCo-ZnAl and PdCu-ZnAl catalysts have a higher surface Pd content than the Pd-ZnAl catalyst. The sample that exhibits the highest surface concentration of palladium is the PdCo-ZnAl and it exhibits the highest Pd/Al surface atomic ratio. These differences in terms of the surface composition of the catalysts can significantly affect the catalytic behavior of the samples under study.

### 3.2. Catatytic Activity

#### 3.2.1. Propene Oxidation

**Figure 4:**
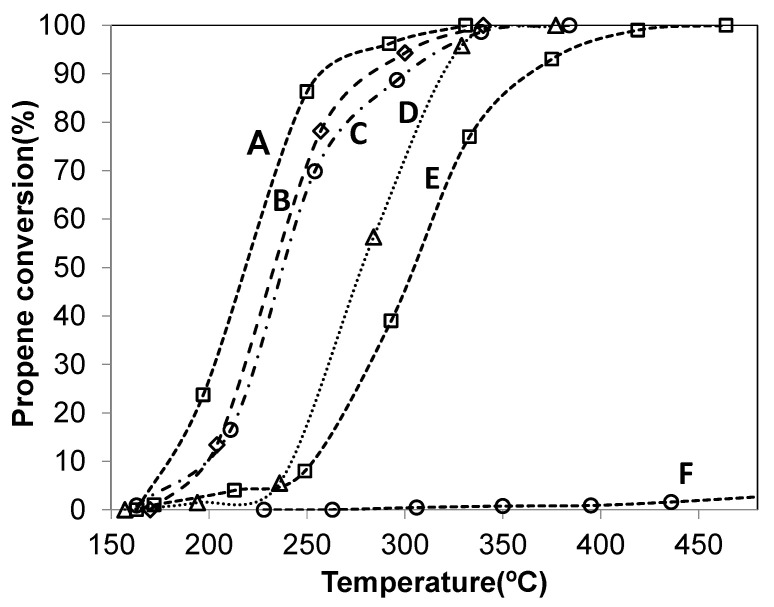
Propene to CO_2_ conversion vs. temperature. (A) PdCo-ZnAl; (B) PdCu-ZnAl; (C) Pd-ZnAl; (D) Co-ZnAl; (E) Cu-ZnAl; (F) without catalyst.

The evolution of propene conversion to CO_2_ as a function of the reaction temperature is shown in Figure 4, for Co-ZnAl (curve D), Cu-ZnAl (curve E) and Pd-containing catalysts (curves A–C). These experiments were carried out using an O_2_/He mixture as oxidizing agent. Table 4 summarizes T_50_ and T_100_ values (temperature at which 50% and 100% conversions are reached, respectively) obtained in each experiment. In the absence of catalyst, propene is poorly oxidized in the O_2_/He atmosphere, reaching 10% conversion at 600 °C, while in the presence of the catalysts this hydrocarbon is completely oxidized in the temperature range 150–500 °C, yielding CO_2_ as the only observed product (~100% of selectivity).

Comparing Co-ZnAl and Cu-ZnAl samples, the Co-ZnAl catalyst was the most active and reached a conversion of 50% at 278 °C (T_50_) (Figure 4, curve D). It is evident that the supported oxide phases, and particularly the cobalt spinel, present the necessary redox properties to promote the oxidation of propene. The Pd-ZnAl catalyst, although it contains a very low concentration of PdO_x_, shows very good activity (Figure 4, curve C). The higher activity of this catalyst can be associated with the greater reducibility of the system, since the monometallic catalyst reduces at a lower temperature.

With respect to the effect of Pd content on activity, the palladium-promoted oxide catalysts presented higher activity than the Co-ZnAl and Cu-ZnAl catalysts, as depicted in Figure 4 and in Table 4. The addition of a small amount of palladium (0.5 wt.%) caused a significant increase in the activity. These results suggest that there is a strong synergism between palladium and cobalt or copper species, all oxides contributing as components of the surface-active phase, as it has been reported for other catalytic formulations that contain an oxide phase and precious metals in their composition [28,36]. The synergic effect of Pd-CoO_x_ or Pd-CuO_x_ can be evidenced by changes both in the nature of the active sites and in their activity.

For hydrocarbons oxidation reactions, the reducibility of the system, the oxygen mobility and the presence of surface defects are factors that might condition the activity. In this work, the synergic effect could be associated with an increase in the reducibility of the catalytic systems for PdCu-ZnAl and PdCo-ZnAl catalysts, as observed in TPR analysis, and an increase in the surface defects for the cobalt-containing sample, which presented a higher Co(II)/Co(III) surface atomic ratio. Besides, the presence of the Co_3_O_4_ phase causes an increase in the PdO_x_ dispersion. TEM micrographs of Pd-ZnAl and PdCo-ZnAl catalysts (Figure S7A) showed particles of PdO_x_ highly dispersed on both catalysts. The histograms of PdO_x_ particle size distribution presented in Figure S7B showed that the supported particles had sizes of between 3 and 9 nm for both Pd-ZnAl and PdCo-ZnAl catalysts. The histogram of the PdCo-ZnAl sample exhibited a higher proportion of smaller particles. The average particle diameter (Dva) calculated from the histograms of particle size distribution was 6.0 nm and 5.4 nm for Pd-ZnAl and PdCo-ZnAl, respectively.

The PdCo-ZnAl catalyst (Figure 4, curve A) presented the highest activity and reached the 50% of propane conversion at 219 °C (T_50_). This sample exhibited some of the characteristics required for oxidation catalysts, high reducibility and surface structural defects. For hydrocarbon oxidation reactions, it was found that the non-ordered or partially ordered cobalt oxide catalysts were more active than the ordered one. In this context, it has been reported that increasing surface disorder produces an increase in the concentration of reactive oxygen defects and, consequently, an increase in the oxygen mobility [56,63,64]. In addition, the PdO_x_ particles are more dispersed on the catalytic surface of this catalyst.

It is important to point out that the materials studied in this work, and particularly the PdCo-ZnAl catalyst, presented high activity, comparable to those reported in the literature [8,65,66,67,68,69,70,71,72,73,74,75,76]. For comparative purposes, a selection of reported catalytic results, in terms of the required temperature to obtain 50% of propene oxidation, is presented in Table 5. It is important to highlight that the catalysts presented in this work contain a low content of noble metal (0.5 wt.%) and show good activity when they are evaluated at an acceptable space velocity (GHSV = 39,500 h^−1^). For example, in a recent work, Li et al. [8] reported that an alumina-supported Pt nanoparticle (NPs) (2.8 wt.% Pt) catalyst showed a very good performance for the propene combustion, with a T_50_ of 250 °C (GHSV = 30,000 h^−1^).

#### 3.2.2. Catalytic Stability for the Propene Oxidation

**Figure 5:**
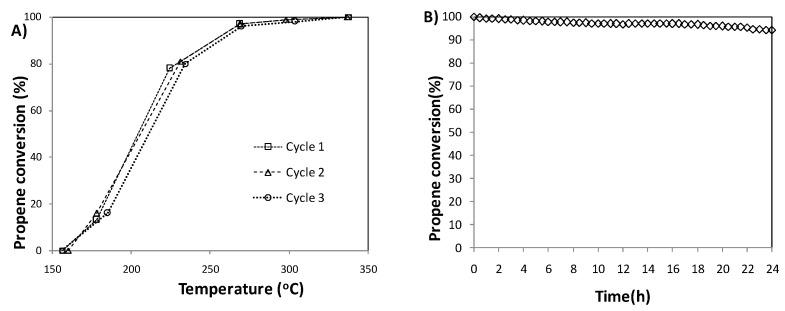
Stability tests for the PdCo-ZnAl catalyst. (**A**) Propane conversion for three successive complete cycles; (**B**) Isothermal stability test at 310 °C for 24 h.

Catalytic stability of PdCo-ZnAl sample was evaluated using two methods. In the first method, the stability was evaluated performing three successive catalytic cycles (Figure 5A), while in the second one, the stability at high conversion values was assessed using an isothermal 24 h experiment, performed at 310 °C (Figure 5B).

No significant temperature shifts were detected in the conversion curves obtained for the three catalytic cycles. Only a slight shift (~10 °C) of the temperatures that correspond to ~18% and ~80% conversion towards higher values were observed. The stability test performed in an isothermal experiment, at high temperature (310 °C), also revealed that no significant activity loss was observed. While the initial conversion is 100%, after 24 h it decreased to 94%. The results obtained suggest that PdCo-ZnAl catalyst exhibit a very good catalytic stability.

**Figure 6:**
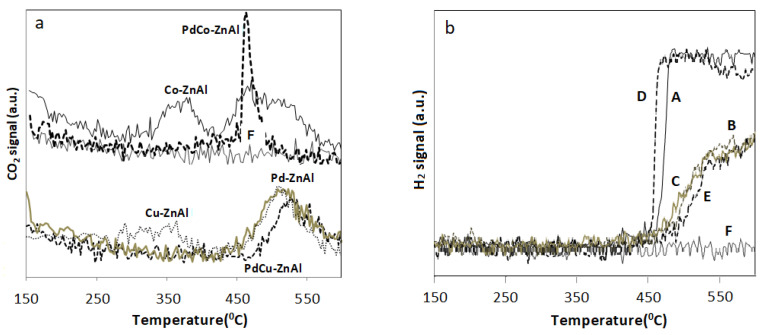
(**a**) CO_2_ signal vs. temperature. (**b**) Signal H_2_ vs. temperature. (A) Co-ZnAl; (B) Cu-ZnAl; (C) Pd-ZnAl; (D) PdCo-ZnAl; (E) PdCu-ZnAl; (F) without catalyst.

Two mechanisms have been proposed to explain hydrocarbons oxidation reactions, one of them is the redox Mars–van Krevelen mechanism, where the catalytic surface provides active oxygen for the reaction, and the other is a Langmuir–Hinshelwood mechanism, which proposes the existence of active adsorption sites where the reactants, hydrocarbons and oxygen species, interact and are transformed into products [77,78]. To analyze some aspects related to the reaction mechanisms that would allow explaining the differences in the catalytic behavior of the catalysts, complementary experiments were carried out with a feed of propene/helium in the absence of O_2_ (g), in a flow reactor *on-line* with a mass spectrometer. These experiments were carried out to analyze if the active phases of catalysts could provide oxygen to oxidize the propene and/or activate it. When experiments are conducted in the absence of molecular oxygen, at increasing temperature CO_2_ is formed (Figure 6a) and the occurrence of a surface redox reaction can be postulated. The CO_2_ produced can only be generated by propene through its reaction with the lattice oxygen of catalysts, following a redox or Mars–van Krevelen mechanism [29,38]. Moreover, it is shown that the catalysts can activate propene and generate H_2_ (g) (Figure 6b). Otherwise, in the absence of a catalyst, neither CO_2_ nor H_2_ are observed.

Likewise, the results illustrated in Figure 6 suggest that the temperature range in which propene activation occurs is strongly influenced by the active species present in the catalysts. Co-ZnAl and Cu-ZnAl catalyst start supplying lattice oxygen at ~250 °C and the CO_2_ evolution vs. temperature curves showed two maxima, the first at ~350 °C and the second one at ~500 °C, indicating the existence of at least two superficial processes. The first one could be associated with a redox mechanism, while the second one, at higher temperature, could be associated with the propene cracking/activation and H_2_ (g) generation, as it is evidenced in Figure 6b. Instead, with palladium-containing catalysts, CO_2_ evolution can be observed at higher temperature (>400 °C).

On one hand, it is evident that the formation of Pd-Co or Pd-Cu interaction structures generates a decrease in the availability of lattice oxygen. Moreover, it is evident that the Mars–van-Krevelen mechanism does not completely explain the high activity of the palladium-containing catalysts at low temperature in the presence of O_2_ (g), because with these systems the total combustion of propene is reached at a temperature lower than 350 °C. Probably, at low temperature, the reaction mechanism of these catalysts involves the adsorption of both the hydrocarbon and the molecular oxygen, following a Langmuir–Hinshelwood type mechanism.

It is interesting to mention that a TPR profile (not shown) of the post-reaction PdCo-ZnAl sample, used in the presence of molecular oxygen, show lower H_2_ consumption at low temperature (<160 °C) than the fresh catalyst. This result suggests that propene could reduce the most accessible active oxide phases during the reaction.

In studies of alkene oxidation reaction mechanisms using metallic catalysts, it has been proposed that the alkene adsorption on the metallic site (e.g., Pt) does not require a previous C–H bond cleavage. The alkene can adsorb via a π bonding between the C=C bond and the metallic atoms. Then, the π-coordinated alkene-metallic species are converted into a di-σ species which leads to C–C bond scission and reaction with adsorbed oxygen [78]. A very strong adsorption of the hydrocarbon could cause a decrease of activity. On the other hand, the importance of the active phase capable of promoting oxygen adsorption should not be discounted and, in this sense, it has been proposed that oxide phases are capable of supplying active oxygen to the metal-oxide interface [6,79]. In this sense, Wan et al. [68] presented a simplified reaction mechanism for the propene oxidation over Pt/BaO/alumina catalysts. In this report, it was proposed that propene adsorbed on the catalytic surface is transformed into carboxilates and formates. Then, these species are oxidized to CO_2_ and H_2_O. The formation of active oxygen species at the Pt−Ba interface produced an increase in the oxidation rate.

**Figure 7:**
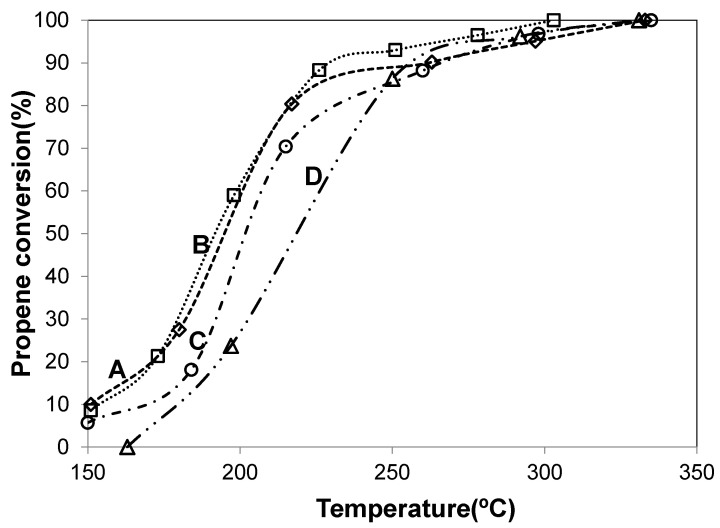
Catalytic activity of H_2_ pre-treated catalysts. (A) Pd-ZnAl_red_; (B) PdCo-ZnAl_red_; (C) PdCu-ZnAl_red_; (D) PdCo-ZnAl.

In this context, it is interesting to study the effect of a mild reducing pretreatment (H_2_ 10% *v/v*, 1 h, 160 °C) on the activity of catalysts. The evolution of the propene conversion with the reaction temperature is shown in Figure 7 and Table 4 summarizes T_50_ and T_100_ values obtained with the pre-treated catalysts.

The H_2_ pre-treated samples showed higher activity than the original catalysts, evidencing the important role of the oxidation state of the species, mainly of the palladium species, on the catalytic activity for the propene combustion. Similar results have been reported with zirconia-supported palladium catalysts [28] and with ceria-supported gold catalysts activated with H_2_ at 300 °C [80].

The PdCo-ZnAl_red_ sample was the most active (Figure 7, curve B). The T_50_ and T_100_ obtained with this catalyst were 191 and 303 °C, respectively.

#### 3.2.3. Catalytic Propane Oxidation

**Figure 8:**
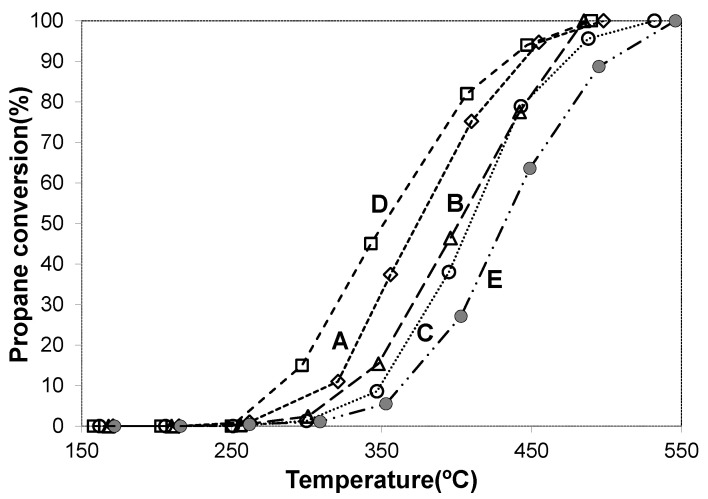
Propane conversion to CO_2_ vs. temperature. (A) PdCo-ZnAl; (B) PdCu-ZnAl; (C) Pd-ZnAl; (D) Co-ZnAl; (E) Cu-ZnAl.

In addition to the very good results found in the combustion of propene, catalytic tests were carried out to evaluate the activity of the catalysts for the total oxidation of propane, a model molecule of a saturated short-chain hydrocarbon. The results obtained are shown in Figure 8. In the absence of catalyst, propane is oxidized to CO_2_, reaching 50% conversion at 600 °C (T_50_). Conversely, all the catalysts are active in the temperature range 250–550 °C, reaching the total oxidation of propane yielding CO_2_ as the only observed product.

For this reaction, the Cu-ZnAl, Pd-ZnAl and PdCu-ZnAl catalysts did not show high activity. Catalysts based on the use of cobalt show higher activity, but for this reaction the addition of palladium does not generate a synergic effect. According to previous reported results [81,82], the palladium-based systems studied in this work did not have high efficiency for propane oxidation. Clearly, this catalyst cannot activate this saturated molecule as effectively as it does with the unsaturated molecule.

## 4. Conclusions

This paper reported the preparation of PdO_x_ and/or MO_x_ ZnAl_2_O_4_-supported catalysts (M = Co, Cu) for the catalytic combustion of propene. The prepared catalysts were examined by using various analytical techniques (BET, XRD, TPR and XPS). It was observed that both transition metals and palladium species are, predominantly, in the form of oxide species. The catalysts were tested for the catalytic combustion of propene and propane. Their activities depended on the nature of the molecule to be oxidized and were influenced by the reducibility of the system. Particularly, the PdCo-ZnAl catalysts exhibited very good performance in the oxidation of propene.

In both the propene and propane combustion reactions, the results obtained showthat Co_3_O_4_–containingcatalysts supported on ZnAlO_4_are more active than those containing CuO.

Noble metal addition to Cu-ZnAl and Co-ZnAl catalysts produces a promoting effect on the propene oxidation. These promoted solid presents high activity and their catalytic behavior is associated with a synergistic effect between palladium species and MO_x_ (M = Co, Cu), which is also evidenced by an increase in the reducibility of the catalytic systems.

## Figures and Tables

**Table 1:** BET surface area (S_g_), pore volume (V_p_) and pore diameter (d_0_) of support and catalysts.

Catalyst	V_p_ (cm^3^/g)	S_g_ (m^2^/g)	d_o_ (nm)
ZnAl	0.280	50	17
Cu-ZnAl	0.274	50	17
Co-ZnAl	0.308	49	16
Pd-ZnAl	0.273	52	15
PdCu-ZnAl	0.276	50	16
PdCo-ZnAl	0.272	42	16

**Table 2:** Nominal, EDS-SEM and XPS-derived surface atomic ratios of catalysts.

Catalyst	EDS			XPS			Nominal		
	Co/Al	Cu/Al	Pd/Al	Co/Al	Cu/Al	Pd/Al	Co/Al	Cu/Al	Pd/Al
Co-ZnAl	0.091	-	-	0.22	-	-	0.082	-	-
Cu-ZnAl	-	0.098	-	-	0.083	-	-	0.076	-
Pd-ZnAl	-	-	0.008	-	-	0.004	-	-	0.004
PdCu-ZnAl	-	0.083	0.006	-	0.032	0.005	-	0.076	0.004
PdCo-ZnAl	0.174	-	0.009	0.17	-	0.006	0.082	-	0.004

**Table 3:** XPS Binding energies (B.E.) of palladium, copper and cobalt species, K.E. of cobalt Auger transitions and Co(II)/Co(III) atomic ratios.

	Cu-ZnAl	PdCu-ZnAl	Co-ZnAl	PdCo-ZnAl	Pd-ZnAl
Pd3*d*_5/2_	-	336.8	-	337.0	336.9
Pd3*d*_3/2_	-	342.3	-	342.6	342.4
Cu2*p*_3/2_	933.7	933.5	-	-	-
sat 2*p*_3/2_	943.1	943.4	-	-	-
Cu2*p*_1/2_	953.3	953.4	-	-	-
Co 2*p*_3/2_	-	-	780.9	781.5	
Co 2*p*_1/2_	-	-	-	-	
sat 2*p*_3/2_ *	-	-	786.6	786.9	
sat 2*p*_1/2_ *	-	-	790.1	790.4	
CoLMM Co(III)	-	-	711.7	711.5	
CoLMM Co(II)	-	-	718.3	717.9	
Co(II)/Co(III)	-	-	0.72	0.76	

* Shake-up satellite.

**Table 4:** T_50_ and T_100_ obtained with the studied catalysts in propene combustion.

Catalyst	T_50_ (°C)	T_100_ (°C)
Pd-ZnAl	242	384
Cu-ZnAl	305	464
Co-ZnAl	278	377
PdCo-ZnAl	219	331
PdCu-ZnAl	232	340
Pd-ZnAl_red_	197	333
PdCu-ZnAl_red_	203	335
PdCo-ZnAl_red_	191	303

**Table 5:** Comparison of deep propene oxidation activity for different catalysts.

Catalyst	T_50_ (°C)	GHSV (h^−1^)	Reference
PdCo-ZnAl	219	39,500	This work
Co-ZnAl	278	39,500	This work
Pt(5)Al_2_O_3_	210	60,000	[68]
Pd(2)/Al-PILC	210	20,000	[69]
Pt(2)/Al-PILC	310	20,000	[69]
Co/M-Clay	302	50,000	[65]
Pt(2.8)γ-Al_2_O_3_	250	30,000	[8]
Pd(1)Al	201	60,000	[67]
Co_3_O_4_-SSgm	300	45,000	[16]
Co_2.1_Fe_0.9_O_4_	348	45,000	[70]
Pd (0.5)/CeO_2_	189	35,000	[6]
Pd(1.5)TiO_2_	226	60,000	[72]
Ir(0.5)Au(1)TiO_2_	285	7800	[73]
Au(1)TiO_2_	242	35,000	[74]
Au(1)Al_2_O_3_	286	35,000	[74]
Pd(0.5)Au(1)TiO_2_	208	60,000	[72]
Au(3.07)Ce_0,3_Ti_0,7_O_2_	242	60,000	[75]
Au(3.7)CeO_2_	170	60,000	[76]

## Data Availability

Data are contained within the article and can be requested from the corresponding author.

## Supplementary Materials

The following are available online at https://www.mdpi.com/article/10.3390/ma14174814/s1, Figure S1: Adsorption/desorption isotherms obtained for the ZnAl support (A) and the PdCo-ZnAl catalyst (B), Figure S2A: SEM micrographs of catalysts, Figure S2B: EDS elemental maps, Figure S3: XPS spectra of support, Figure S4: Pd 3d XPS spectra of catalysts. (A) Pd-ZnAl; (B) PdCu-ZnAl; (C) PdCo-ZnAl, Figure S5: Co 2p XPS spectra and deconvolution. (A) Co-ZnAl catalyst; (B) PdCo-ZnAl catalyst, Figure S6: Cu 2p XPS spectra of catalysts. (A) Cu-ZnAl; (B) PdCu-ZnAl, Figure S7A: TEM micrographs of catalysts. (a) ZnAl support; (b) Pd-ZnAl and (c) PdCo-ZnAl, Figure S7B: Histograms of PdOx particle size distribution. (a) Pd-ZnAl and (b) PdCo-ZnAl.
