# Supplementary material for: Promoting Effect of Palladium on ZnAl2O4-Supported Catalysts Based on Cobalt or Copper Oxide on the Activity for the Total Propene Oxidation
Source: Materials (Basel). 2021 Aug 25;14(17):4814. doi: 10.3390/ma14174814 (PMC8432500; doi:10.3390/ma14174814)
Supplement: Supplementary file 1 [file materials-14-04814-s001.zip › materials-1332250-Supplementary Materials.pdf]

materials

MDPI

Supplementary Material

Promoting effect of palladium on $ZnAl_2O_4$-supported catalysts based on cobalt or copper oxide on the activity for the total propene oxidation.

Marco Antonio Ocsachoque, María Silvia Leguizamón-Aparicio, Mónica Laura Casella and Ileana Daniela Lick*

CINDECA (CCT La Plata-CONICET-UNLP), Departamento de Química, Facultad de Ciencias Exactas, Universidad Nacional de La Plata, Calle 47 N° 257, La Plata, Buenos Aires 1900, Argentina; ocmarco@quimica.unlp.edu.ar (M.A.O.); mariasilvialap@quimica.unlp.edu.ar (M.S.L.-A.); casella@quimica.unlp.edu.ar (M.L.C.)

* Correspondence: ilick@quimica.unlp.edu.ar; Tel.: +54-221-4211353

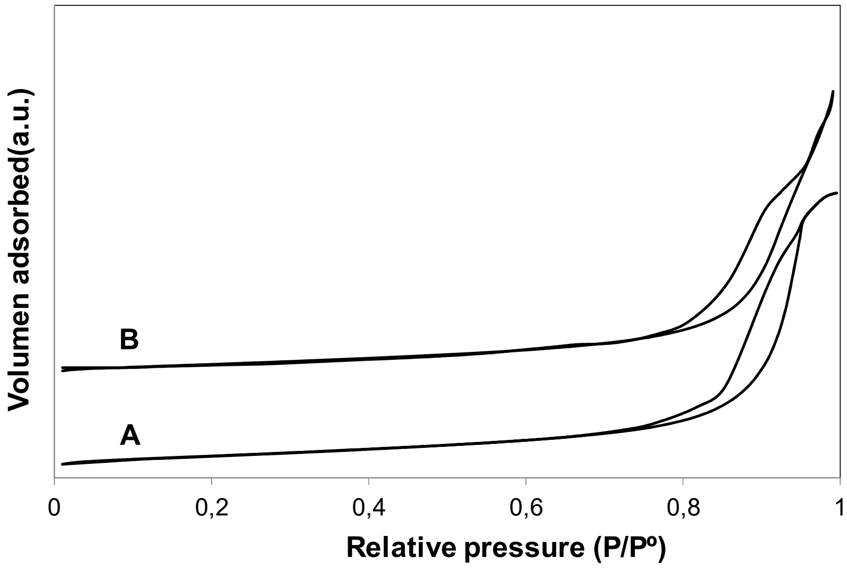

Figure S1. Adsorption/desorption isotherms obtained for the ZnAl support (A) and the PdCo-ZnAl catalyst (B).

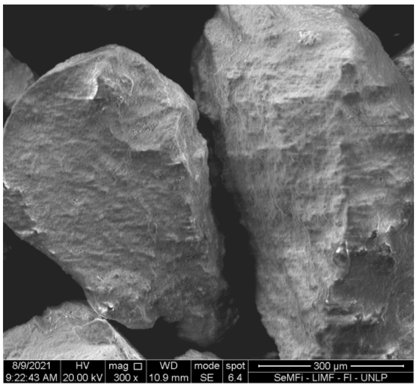

a) Co-ZnAl

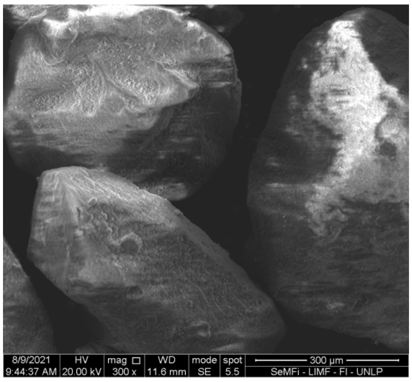

b) PdCo-ZnAl

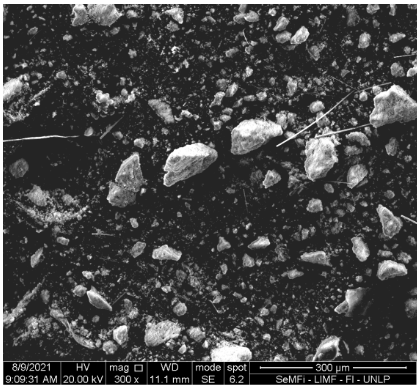

c) Cu-ZnAl

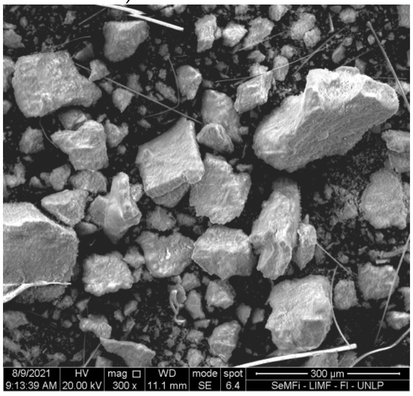

d) PdCu-ZnAl

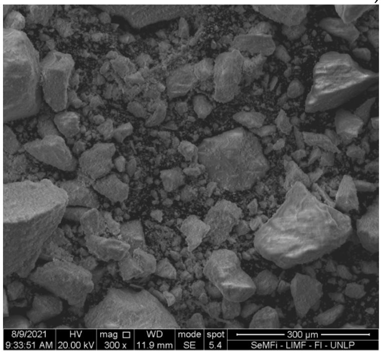

e) Pd-ZnAl

Figure S2A. SEM micrographs of catalysts.

a) Co-ZnAl Catalyst (300X)

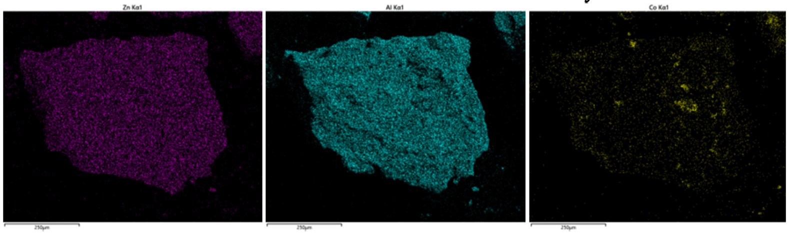

Zn Al Co

b) Co-ZnAl Catalyst (5000X)

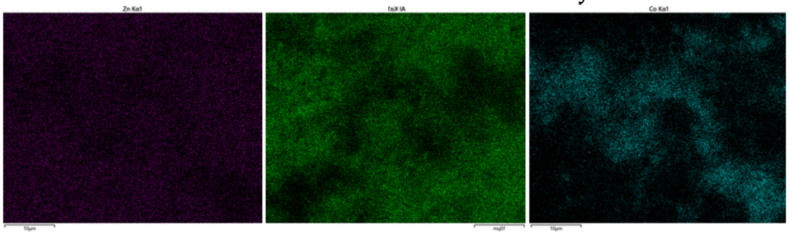

Zn Al Co

c) PdCo-ZnAl Catalyst (300X)

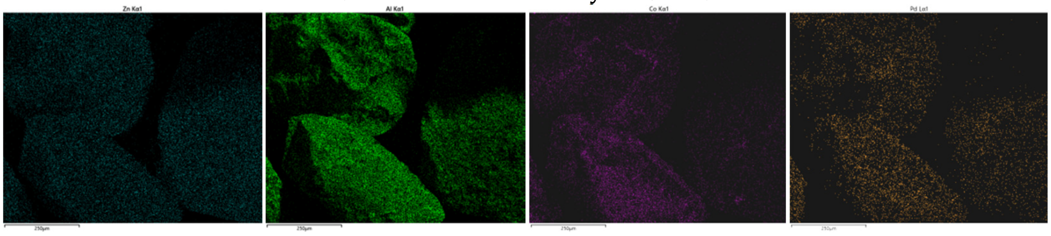

Zn Al Co Pd

d) PdCo-ZnAl Catalyst (5000X)

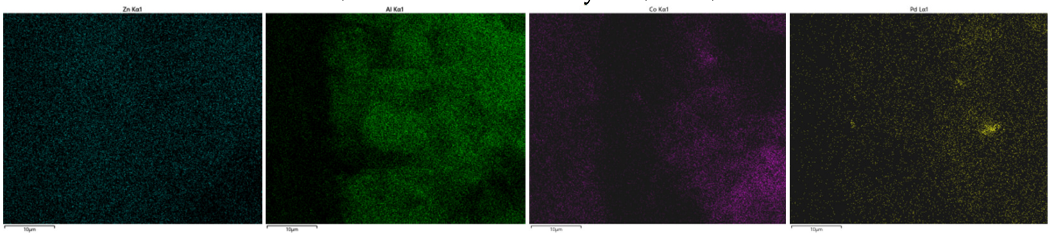

Zn Al Co Pd

e) Cu-ZnAl Catalyst (300X)

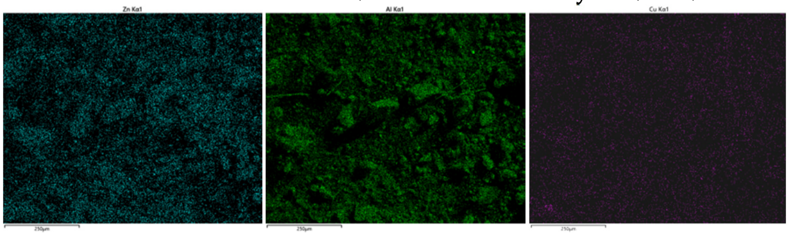

Zn Al Cu

f) Cu-ZnAl Catalyst (5000X)

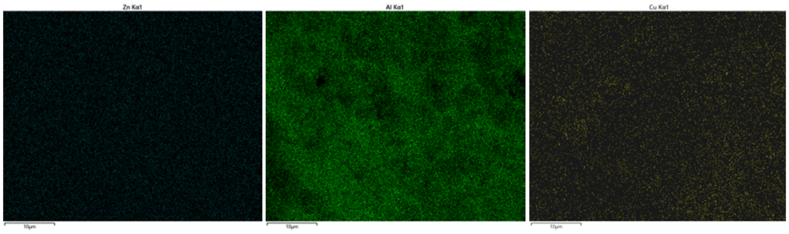

Zn Al Cu

g) PdCu-ZnAl Catalyst (300X)

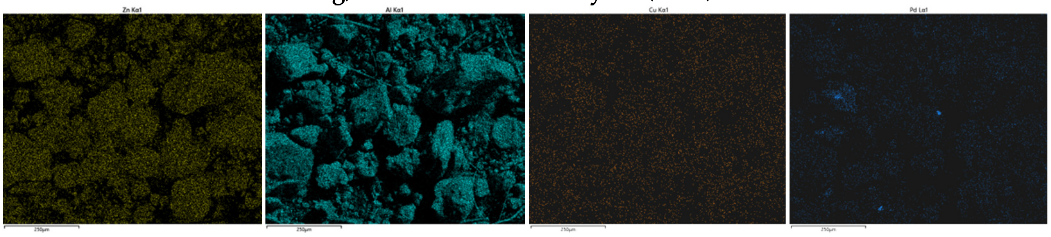

Zn Al Cu Pd

h) PdCu-ZnAl Catalyst (5000X)

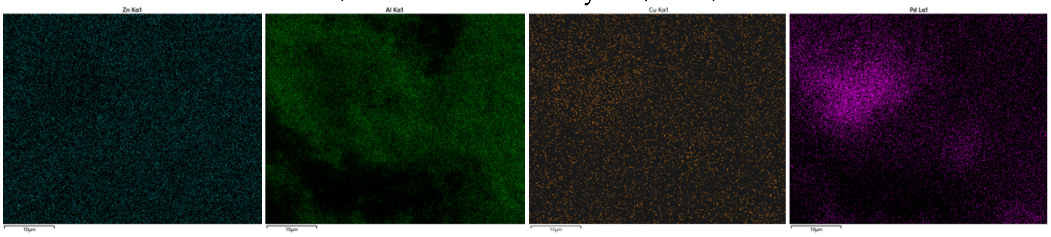

Zn Al Cu Pd

i) Pd-ZnAl Catalyst (300X)

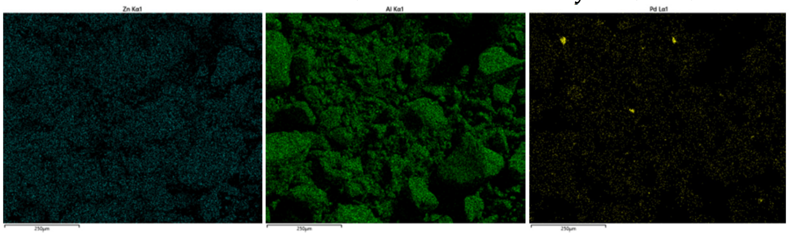

Zn Al Pd

j) Pd-ZnAl Catalyst (5000X)

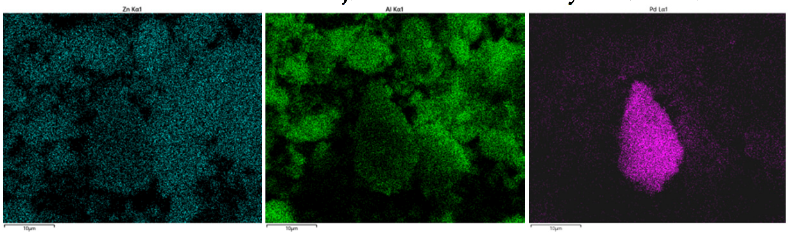

Zn Al Pd

Figure S2B. EDS elemental maps.

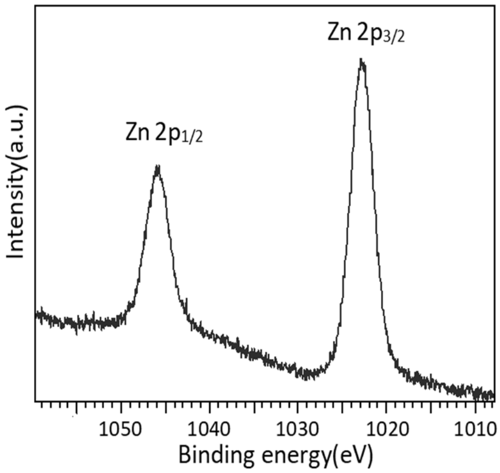

Zn 2p

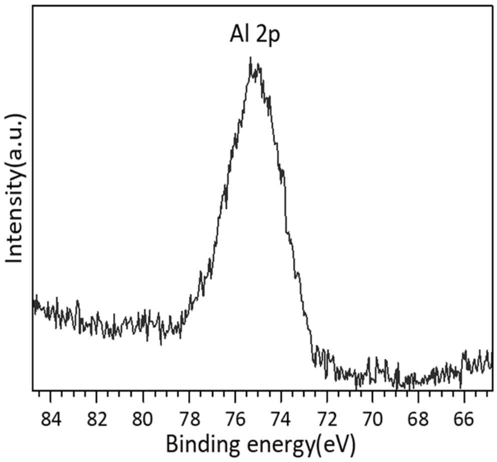

Al2p

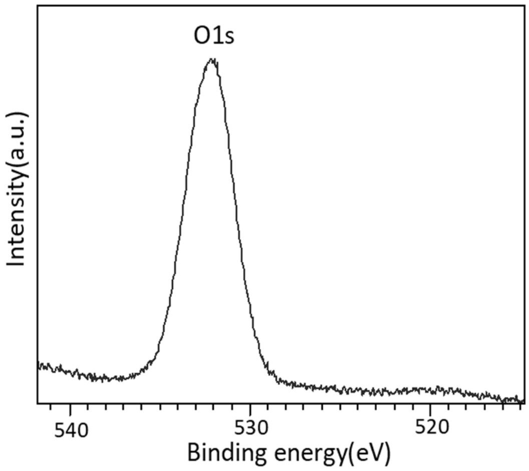

O 1s

Figure S3. XPS spectra of support.

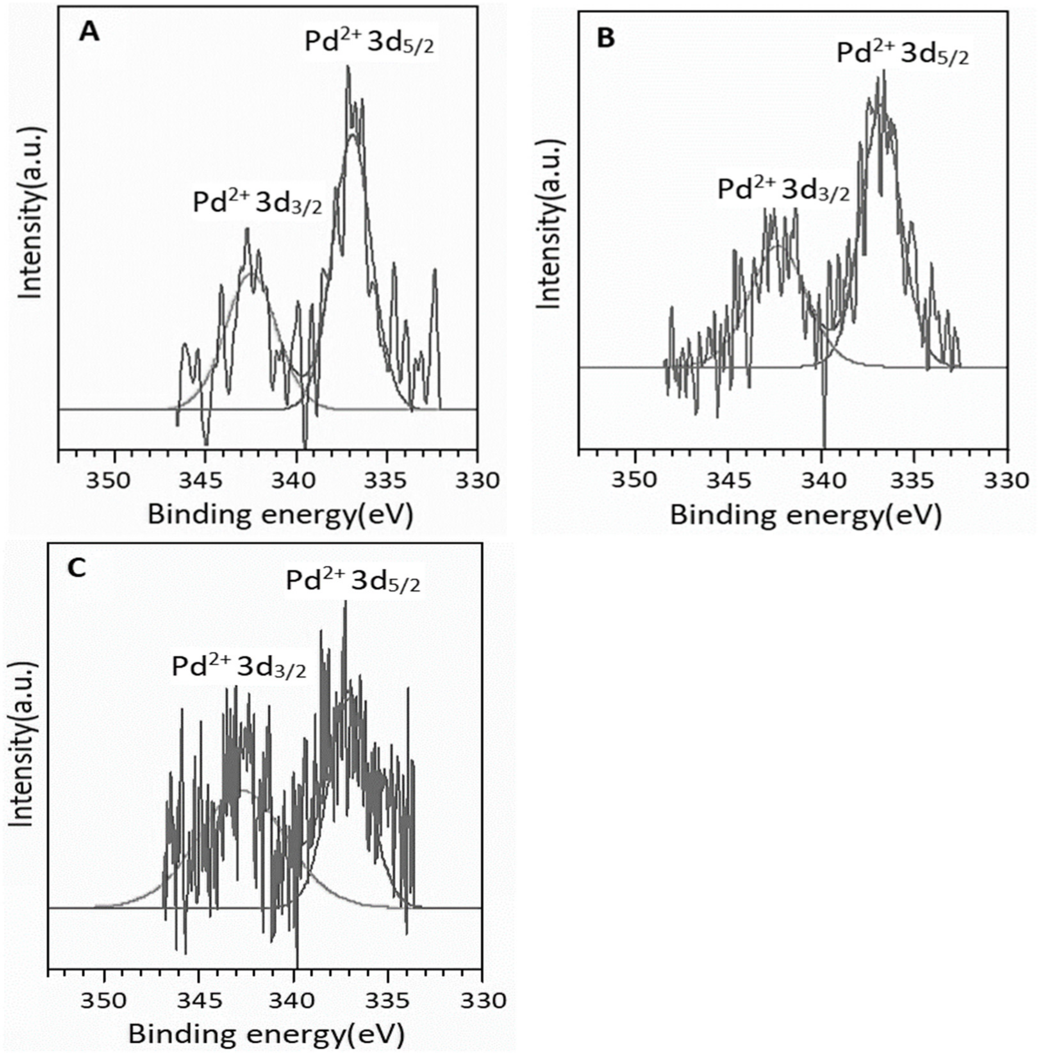

Figure S4. Pd 3d XPS spectra of catalysts. A) Pd-ZnAl; B) PdCu-ZnAl ; C) PdCo-ZnAl.

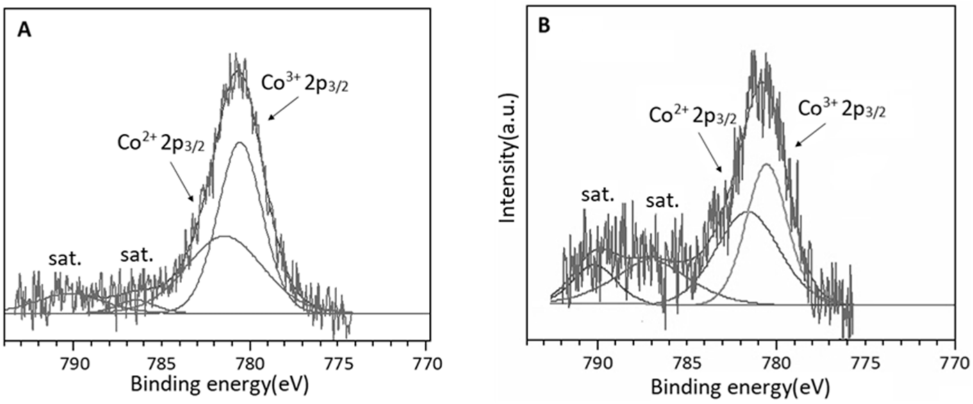

Figure S5. Co 2p XPS spectra and deconvolution. A) Co-ZnAl catalyst; B) PdCo-ZnAl catalyst.

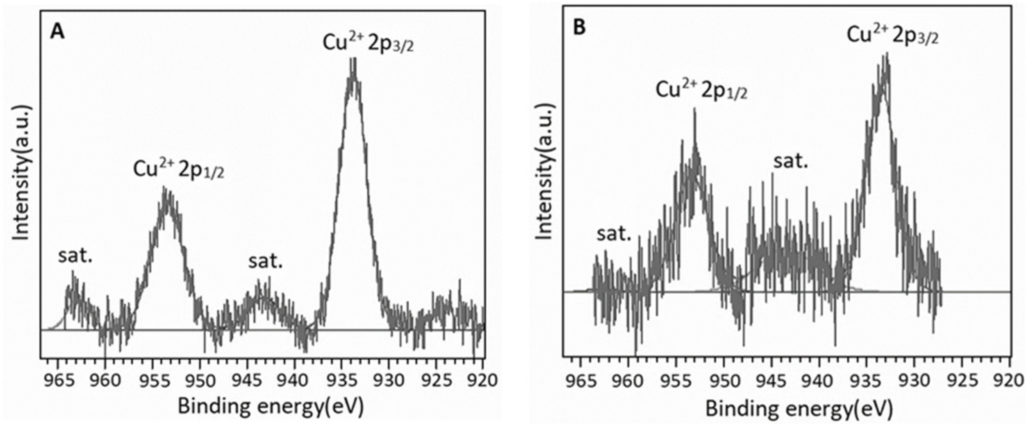

Figure S6. Cu 2p XPS spectra of catalysts. A) Cu-ZnAl; B) PdCu-ZnAl.

a) **b)** **c)**

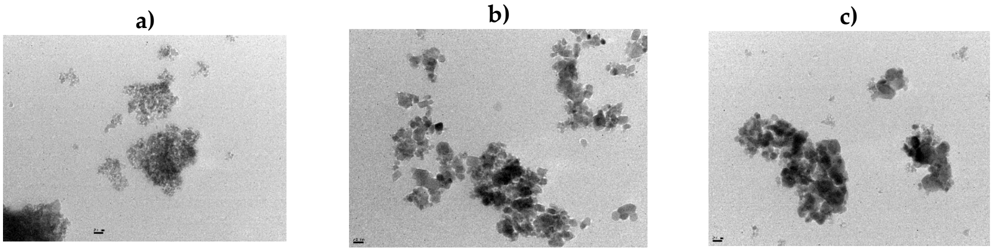

Figure S7A. TEM micrographs of catalysts. a) ZnAl support, b) Pd-ZnAl and c) PdCo-ZnAl.

a) b)

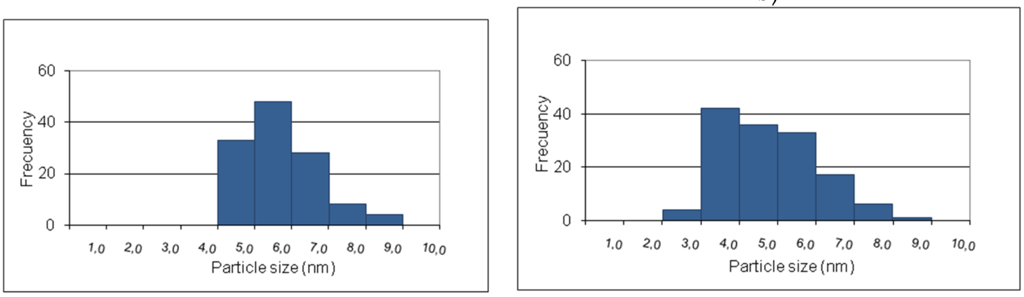

Figure S7B. Histograms of PdOx particle size distribution. a) Pd-ZnAl and b) PdCo-ZnAl.
